# The Influence of Cement Type on the Properties of Plastering Mortars Modified with Cellulose Ether Admixture

**DOI:** 10.3390/ma14247634

**Published:** 2021-12-11

**Authors:** Edyta Spychał, Przemysław Czapik

**Affiliations:** Faculty of Civil Engineering and Architecture, Kielce University of Technology, 25-314 Kielce, Poland; p.czapik@tu.kielce.pl

**Keywords:** cellulose ether, cement plastering mortar, mineral additives, consistency, bulk density, water retention, cement paste hydration, flexural and compressive strength

## Abstract

In this article, the effect of cement type on selected properties of plastering mortars containing a cellulose ether admixture was studied. In the research, commercial CEM I Portland cement, CEM II and CEM III, differing in the type and amount of mineral additives, and cement class, were used as binders. Tests of consistency, bulk density, water retention value (WRV), mechanical properties and calorimetric tests were performed. It was proved that the type of cement had no effect on water retention, which is regulated by the cellulose ether. All mortars modified with the admixture were characterized by WRV of about 99%. High water retention is closely related to the action of the cellulose ether admixture. As a result of the research, the possibility of using cement with additives as components of plasters was confirmed. However, attention should be paid to the consistency, mechanical properties of the tested mortars and changes in the pastes during the hydration process. Different effects of additives resulted from increasing or decreasing the consistency of mortars; the flow was in the range from 155 mm to 169 mm. Considering the compressive strength, all plasters can be classified as category III or IV, because the mortars attained the strength required by the standard, of at least 3.5 MPa. The processes of hydration of pastes were carried out with different intensity. In conclusion, the obtained results indicate the possibility of using CEM II and CEM III cements to produce plastering mortars, without changing the effect of water retention.

## 1. Introduction

Human activities have an increasing impact on the surrounding natural environment. Reduction of CO_2_ production has long been the main problem of the global economy, presenting challenges in areas such as engineering, environmental protection and the construction industry [1,2,3,4,5,6,7,8,9,10,11,12]. CO_2_ emissions from fossil fuels and industry account for approximately 90% of all CO_2_ emissions into the atmosphere from human activity. Cement production alone accounts for about 5% of global CO_2_ emissions [1]. CO_2_ emissions computed for the finished cement depend mainly on the clinker content, especially for CEM I cement [5]. The reduction of carbon dioxide emissions from cement production is, therefore, an important and urgent task for the cement industry. One of the possible ways to limit the use of clinker is the use of cement with a high content of mineral additives [4,6,10,11,12,13]. Siliceous fly ash, calcareous fly ash and granulated blast-furnace slag are traditionally used in the production of cement. These additives have pozzolanic and hydraulic properties, respectively, which advantageously influence cement properties. The use of these raw materials in the production of cement thus reduces carbon dioxide emissions [7,8,9,10,12].

Cement is one of the most popular binders used in dry-mix mortars, such as plastering mortars, masonry mortars and adhesive mortars [14,15,16,17]. Cement in these materials acts as a binder ensuring obtaining the appropriate strength class and the durability of the finished product. It is also largely responsible for the adhesion of the mortar to the substrate [15,17]. Portland cement CEM I is the basic binder in mortars, but more often this cement is replaced by CEM II multi-component and CEM III [17].

Modern plastering mortars are complex multi-component systems. Among the mortar components (besides binder, fine aggregate and water), cellulose ether admixture plays an important role in dry-mix mortars [18,19,20,21,22,23,24,25,26,27]. Cellulose ethers as polymer admixtures are being applied to a growing extent in the production of dry-mix mortars. This leads on the one hand to a great variety of areas of application and on the other hand to an increasing diversity of mortars. First of all, these polymers improve water retention [18,19,21,22,23,25]. Their function is to prevent water loss into porous, absorbent substrates [23]. High water retention provides proper conditions for the binding and hardening processes of a binder [26], and this ability has a positive effect on reducing mortar shrinkage [27,28,29]. Cellulose ethers have a significant impact on the rheology of fresh mortars [19,21,30,31]. 

In article [14], Chłądzyński assessed the suitability of cements with additives as a binder used in the production of adhesive mortars. The subject of the research was mortars prepared from CEM I Portland cements of various specific surface area and mortars with multi-component Portland cements CEM II (containing varying amounts of fly ash and granulated blast-furnace slag). Cements made in the laboratory were used for the tests, through joint grinding clinker, gypsum, silica fly ash or granulated blast-furnace slag. All samples contained a constant amount of cellulose ether and redispersible powder. Standard tests of physical and mechanical properties of cements were performed, as were calorimetric tests of the heat of hydration and standards tests of adhesive mortars. The results of tests of adhesive mortars with fly ash differed from the results obtained in the case of Portland cement mortars. The effect of fly ash addition was different for individual methods. On the one hand, the research showed slightly better results in terms of adhesion after thermal ageing, but on the other hand, the addition lowered the adhesion values under sample conditions (adhesion tests after immersion of samples in water, adhesion tests due to freeze-thaw cycles). Adhesive mortars made of cement with fly ash show smaller slip versus CEM I Portland cement mortars. The effect of granulated blast-furnace slag addition in adhesive mortars was similar to the effect of fly ash. The addition of granulated blast-furnace slag also improves the open time for tested mortars. As a result of the research, it was found that the tested cements with additives can be used as a binder in the composition of adhesive mortars. The influence of cement replacement by fly ash in brick masonry strength was experimentally verified by Seshu and Murthy in their article [32]. The research consisted of the casting and testing of brick masonry prisms, with two bricklayers. Cement and cement-fly ash mortars were prepared. In each mix the fly ash percentage replacing cement binder in the mortars was increased from 0% to 40%, in intervals of 10%. The results showed that replacement of cement with fly ash in cement mortars is possible up to 40%, without unfavorable effects on the properties of the masonry mortars. The tested additive replacement in leaner cement mortar mixes resulted in the loss of mechanical properties by more than 15%, so cement replacement with fly ash, in this case, may be not useful or profitable. Mortars containing cement and fly ash modified with chemical admixtures have been researched by Zhou and et.al. [33]. All samples contained a constant amount of cement and additive, but the amount of cellulose ether, starch ether, bentonite and redispersion emulsoid powder were variable. The research was an evaluation of the consistency, water retention, setting time, compressive strength, but the effect of the fly ash on the properties tested was not analyzed. The authors focused on the evaluation of the working and mechanical properties of ordinary dry-mixed mortars. It was found that cellulose ether admixtures had the biggest influence on the consistency, water retention and compressive strength of mortars, among all the analyzed chemical admixtures. 

This paper describes how the type of cement affects the plastering mortars’ selected properties, i.e., consistency, water retention, flexural and compressive strength and hydration process. The described experimental results constitute the first part of our research, concerning the assessment of the suitability of cements CEM II and CEM III as a binder in plastering mortar modified with cellulose ether admixture. The scope of further planned research is presented in the conclusions of this article. The research conducted so far has focused mainly on the use of cement CEM I, hydraulic lime or hydrated lime for the properties of the plasters. This article may be a supplement to the knowledge on the interaction of cellulose ether with cements containing additives. Nowadays, the use of additives in the production of cements is an important issue from the point of view of sustainable development, ecology and economic considerations. The goal of the investigation was the assessment of the suitability of the chosen cements CEM II and CEM III as binders in cement-based plastering mortars modified with cellulose ether—to determine the influence of these binders on the selected functional and mechanical properties of plastering mortars. Additionally, in order to complete the tests of flexural and compressive strength of mortars, calorimetric measurements of pastes were performed.

## 2. Materials and Methods

### 2.1. Materials and Sample Preparation

Commercial bag cement CEM I, CEM II and CEM III (from various cement plants), quartz sand 0.5–1.4 mm (Kreisel, Dąbrowa, Poland), cellulose ether admixture (WALOCEL, The Dow Chemical Company, Midland, MI, USA) and tap water were used. Cellulose ether used in tests is a hydroxyethyl methyl cellulose (HEMC) with the viscosity of 25,000 mPa·s. This admixture is in the form of white powder and it has a low level of chemical modification. Five main types of mortars were prepared for the tests. The first type of mortar (C1) was the reference one, which was prepared using an ordinary Portland cement, CEM I 42.5R, with cellulose ether admixture. The remaining mortars were prepared based on CEM III/A 32.5 N-LH, CEM II/B-V 42.5 R, CEM II/B-M (V-LL) 32.5 R, CEM II/B-V 32.5 R cements, marked sequentially as C2, C3, C4 and C5. All cements met the requirements of EN 197-1 standard. In addition, in the case of selected properties, the C0 mortar was prepared using CEM I 42.5 R cement and did not contain a cellulose ether admixture. The chemical composition and selected physical and mechanical properties of cements obtained from the cement plants are presented, respectively in Table 1 and Table 2.

All samples were prepared and tested in an air-conditioned laboratory at the temperature of 20 ± 2 °C and at a relative humidity of 65 ± 5%. 

The mortar mix proportion is detailed in Table 3. The samples were made with a binder to fine aggregate weight ratio 1:3. The water to binder ratio was 0.7 for all mortars. The amount of water was selected in such a way that the C1 mortar had a flow of 165 mm (consistency within borders 175 ± 10 mm). All samples from C1 to C5 contained a constant amount of cellulose ether admixture, in quantity 4 g. The amount of the admixture was selected experimentally and based on the analysis of the literature [18,19,20,21,26,31].

### 2.2. Methods

The measurements of standard consistency were done according to PN-EN 1015-3:2000 + A2:2007 [34] and PN-B-04500:1985 standards [35].

The bulk density of fresh mortars was determined in accordance with PN-EN 1015-6:2000 + A1:2007 standard [36], but the bulk density of hardened mortars was determined in accordance with PN-EN 1015-10:2001 + A1:2007 standard [37].

Water retention value was determined in the accordance with the defined guidelines [38]. These tests were performed after 10, 30 and 60 min and were defined as WRV10, WRV30 and WRV60. This parameter was determined by weighing absorbent materials (filter paper) placed on the fresh sample before and after the predetermined measurement time. Water retention was calculated according to the formula [38]:

(1) WRV=100−W3 [%]

(2) $$W3=\frac{W2}{W1}\cdot 100\;\left[\%\right]$$

In Formula (1), W3 means the relative water loss in the mortar, expressed as a percentage. In Formula (2), W2 means water mass absorbed by the filter paper, but W1 means water content in the tested mortar in the plastic ring (expressed in grams) [38].

The flexural strength and compressive strength of cement mortars were determined in accordance with PN-EN 1015-11:2001 + A1:2007 standard [39]. For each mortar, three cuboid samples of mortar of 40 mm × 40 × mm × 160 mm dimensions were prepared. Mechanical properties measurements were performed after 2, 7 and 28 days.

Samples intended for testing properties of hardened mortars (bulk density and mechanical properties), after their disassembly (2 days after preparation), were stored for 5 days in polyethylene bags, and then for another 21 days in dry air conditions.

The hydration heat evolution of cement pastes was investigated using a differential conducting microcalorimeter at 20 °C for 72 h. The pastes were prepared as mixtures of 4.5 g of cement, 3.15 g of water and 0.04 g of admixture. The w/c ratio of all samples was 0.7. The research used the BT2.15CS low-temperature differential scanning microcalorimeter (Setaram, Plan-Ies-Ouates, Geneva, Switzerland) operating under non-isothermal and non-adiabatic conditions.

## 3. Results

### 3.1. Consistency Measurements

In Table 4, the results of the consistency for all samples are presented (measurements made with the flow table method in mm and measurements made with the drop cone in cm). 

The flow of C1 mortar was 165 mm. This value was established as the baseline. All mortars from C1 to C5 are characterized by plastic consistency, according to the standard PN-EN 1015-3:2000+A2:2007 [34] (flow diameter in the range from 140 mm to 200 mm) [36,40]. The lowest flow among mortars modified with admixture was observed with C4 mortar (155 mm), but the largest was observed with C2 mortar (169 mm). In both cases the type of additive influenced the consistency. Ground granulated blast-furnace slag increases the flow of the mortars, while the use of limestone increases the water demand of mortars, thus reducing their flow. A similar trend can be observed in the case of the cone penetration test (consistency test according to the standard PN-B-04500:1985). Taking into account the results of consistency of C1–C5 mortars in accordance with [35], it can be concluded that all tested materials achieve the consistency value characteristic of typical plasters used in practice [38,40]. In the case of plastering mortars intended for manually applied plasters, their consistency (according by PN-B-04500:1985 standard) should be 6–9 cm, while for mechanical (by machine) application it should be 8–11 cm [38]. All mortars modified with cellulose ether admixture can be applied manually. Only C1 and C2 mortars can be applied by a machine. 

**Figure 1:**
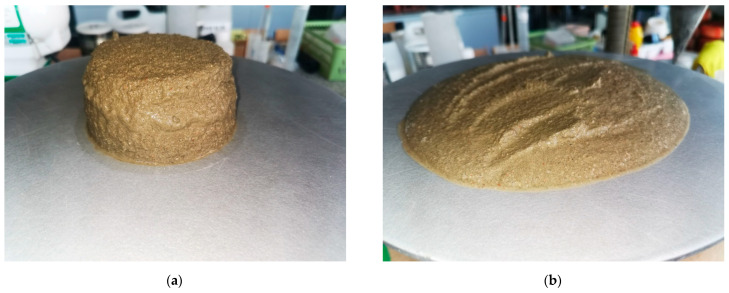
(**a**) View of the C0 sample after removing the form for flow research; (**b**) View of the C0 sample after flow test.

Table 4 shows the results of mortar consistency tests without admixture (sample marked with the symbol C0). It is clearly visible that the mortar without admixture has the greatest consistency in comparison to mortars modified with cellulose ether (these differences vary from 18% to even 35%). Cellulose ether significantly reduces the consistency. Mortars containing this admixture in their composition are characterized by good workability, no segregation of ingredients, which can be seen when comparing the appearance of the tested materials—flow test (Figure 1a,b).

**Figure 2:**
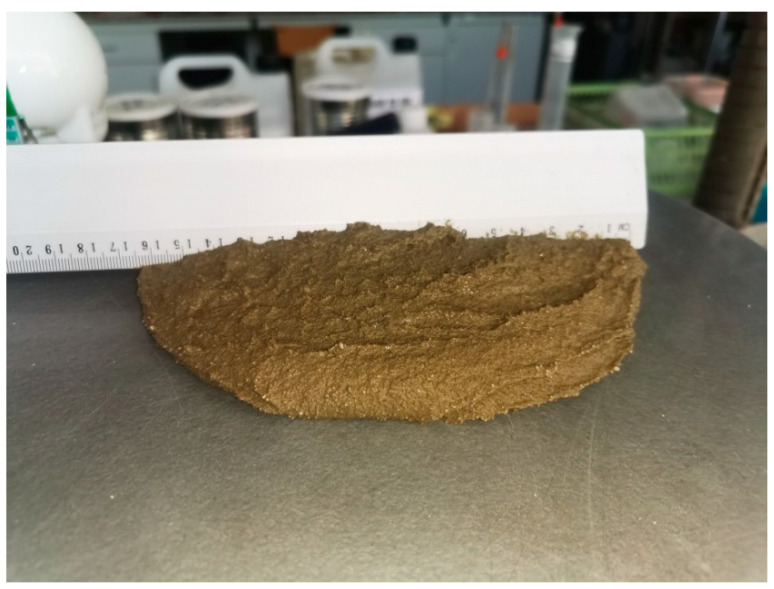
View of the C1 sample after flow test.

Figure 1a,b shows the appearance of the C0 sample during the flow test. Even before the final measurements are taken, water is separating immediately after removing the mold. After measuring the flow diameter, one can also see water separating from the sample. This phenomenon is not observed in the case of other materials. Figure 2 shows the appearance of a mortar sample with CEM I cement and an admixture. The mortar is consistent, there are no visible signs of segregation of ingredients. The consistency measurements thus confirm the advantages of using cellulose ether admixtures, which improves the rheological and application properties of plastering mortars. 

### 3.2. Water Retention

**Figure 3:**
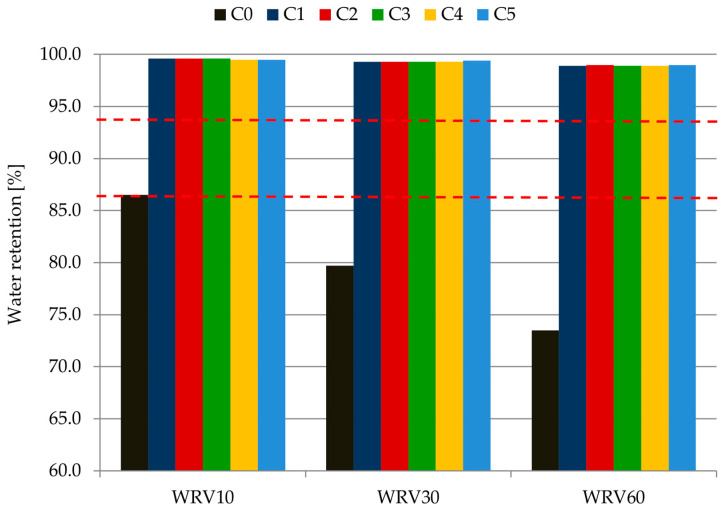
Change in water retention value of mortars C0–C5 over time.

Table 5 and Figure 3 present the results of the water retention values WRV10, WRV30 and WRV60 (the tests were made after 10, 30 and 60 min measurements). 

Based on the research, it can be concluded that all mortars modified with cellulose ether admixture are characterized by a high water retention value throughout the whole test. Changes in water retention during the 60 min of the measurement are practically imperceptible (within 1%). Mortars from C1 to C5 can be classified according to the classification given by Brumaud et al. [22] as materials with high water retention (WRV > 94%), but mortar C0 has low water retention (WRV < 86%). A high water retention level is marked with a solid line in the Figure 3. A high water retention for plasters C1–C5 is related to the action of the admixture. Cellulose ether impacts the viscosity of mortar and causes greater water retention [18,19,21,41]. A part of the water is bonded in the first stage of cement hydration. At the same time, the remaining amount of water forms a gel with the admixture. In this gel, the water molecules are attracted by the functional groups from the polymer and agglomeration process takes place. As the hydration process occurs, this gel can release water into the system [26]. These conclusions also confirm the results obtained for mortar C0. The water retention value for this sample differed significantly from the others; moreover, it underwent changes over time. After 10 min, retention was 86.5% and after 60 min it was 73.5%. Water loss for mortar C0 was thu 26.5%, while it was a maximum of 1.1% for all the modified mortars. In conclusion, there is no apparent influence of type of cement on the water retention value and the change in water retention over time. 

### 3.3. Bulk Density Measurements

**Figure 4:**
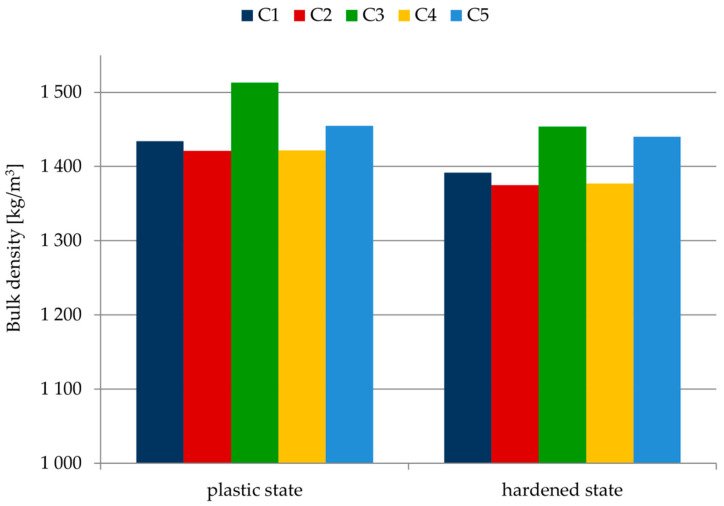
Bulk density results of mortars in plastic and hardened states.

Table 6 and Figure 4 present the results for the bulk density for mortars in the plastic and hardened state. 

The bulk density of fresh mortars is different. The parameter ranges from 1421 kg/m^3^ (C2 mortar) to 1513 kg/m^3^ (C3 mortar). The results for the bulk density of four of the tested samples are within the limits 1421–1455 kg/m^3^, while the bulk density of the C3 sample differs from the others and amounts to 1513 kg/m^3^. The plaster performance can be indirectly assessed on the basis of the parameters affecting the application properties of mortars (ease of application on the substrate, processing time) [19,21,26]. Taking into account the obtained results, mortars C2 and C4, are characterized by the biggest efficiency. Use of these plasters would be the most advantageous in terms of economy (bigger efficiency—lower costs related to material consumption) [21]. Due to the obtained results for bulk density (≥1300 kg/m^3^), the tested plasters are defined as ordinary mortars [40]. When it comes to the results for mortar bulk density in the hardened state, these range from 1375 kg/m^3^ to 1454 kg/m^3^. The lowest bulk density in the plastic and hardened state was achieved by C2 and C4 mortars. Mortar C3 with CEM II/B-V 42.5 R cement obtained the highest bulk density.

### 3.4. Results of Mechanical Properties

**Figure 5:**
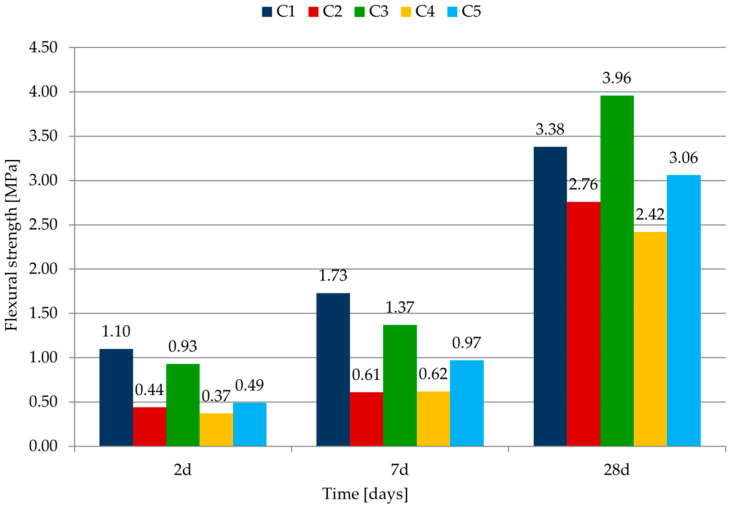
Change of flexural strength after different curing times.

The strength measurements were done after 2, 7 and 28 days of curing. The values from three bars (flexural strength) or six bars (compressive strength) were calculated as an average. The results for flexural strength are shown in Table 7 and in Figure 5. 

Mortar C1 with cement CEM I 42.5 R (cement without addition) is characterized by the highest strength after 2 and 7 days of maturation. The early strength of the mortars C2–C5 was lower than that of the reference sample C1—the difference after 2 days of maturing was in the range of 15% to 66%. This was a result of the type of binder (class of cement and type of addition). The use of cement CEM II/B-V 42.5 R as cement CEM I 42.5 R replacement brings about strength increase at a later age. Mortar C3 (with cement CEM II/B-V 42.5 R) has the highest strength after 28 days. 

**Figure 6:**
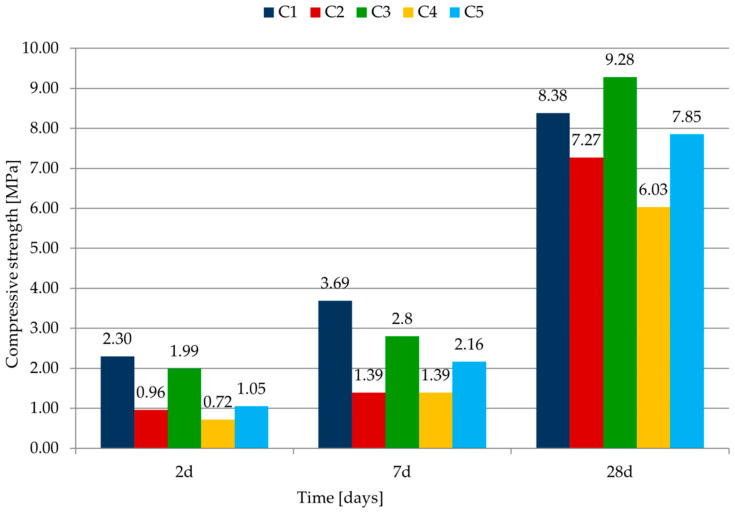
Change of the compressive strength after different curing times.

The results of compressive strength are shown in Table 7 and in Figure 6. 

The results of the compressive strength tests are similar to the results of the flexural strength tests. Mortar with cement CEM I 42.5 R is characterized by the highest strength after 2 and 7 days of maturation. This is due to the lower content of Portland cement clinker in CEM II and CEM III. Mortar with CEM II/B-V 42.5 R is characterized by the highest compressive strength after 28 days. Mortar with this cement has a bigger strength than the base mortar, made of cement without additives. Similar conclusions from the research were obtained in [32]. The authors concluded that fly ash as partial replacement of cement is very useful in mortar with high cement content. 40% replacement is possible without much affecting the strength of the mortars. 

As one could expect, mortars with the cement of class 42.5 of high early strength (C1 and C3) are characterized by the highest flexural and compressive strength after 28 days, regardless of the type of cement, due to the additives. 

Comparing C4 and C5 mortars with CEM II cement, differing in the type of additives, it can be concluded that the strength of mortars with fly ash is only greater than the results for mortars with fly ash and limestone (during the study period). 

According to the classification of plastering mortars included in the PN-EN 998-1:2012 standard [42], all mortars can be classified as categories III and IV due to the compressive strength after 28 days. 

**Figure 7:**
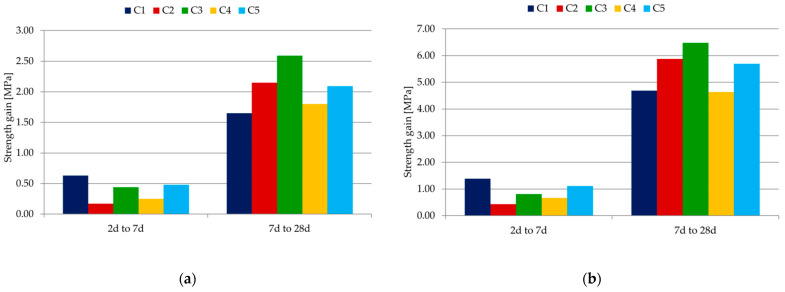
(**a**) Flexural strength gain over time. (**b**) Compressive strength gain over time.

Table 8 and Table 9 show a comparison of the strength in relation to the reference mortar (C1); the results are given as a percentage. Changes in the increment of flexural and compressive strength in MPa were also determined, relating the strength results obtained after 7 and 28 days to the test results after 2 days of specimen maturation. Figure 7a,b shows the gain of flexural and compressive strength over time.

The biggest increase in strength after 7 days was recorded for C1 mortar with CEM I 42.5 R cement, and the lowest for C2 mortar with CEM III/A 32.5 N-LH cement, in which the largest amount of Portland clinker is replaced by a mineral additive in the form of ground granulated blast-furnace slag. Other results can be seen when comparing the strength gains after 28 days. The biggest increase in strength after 28 days was recorded for C3 mortar with CEM II/B-V 42.5 R cement, and the lowest for C4 mortar with CEM II/B-M (V-LL) 32.5 R cement. Cements with chemically active mineral additives allow us to obtain significantly higher strength. However, in the case of the C4 sample, this effect is significantly reduced by the use of a chemically inactive additive—limestone.

### 3.5. Heat of Hydration for Pastes

**Figure 8:**
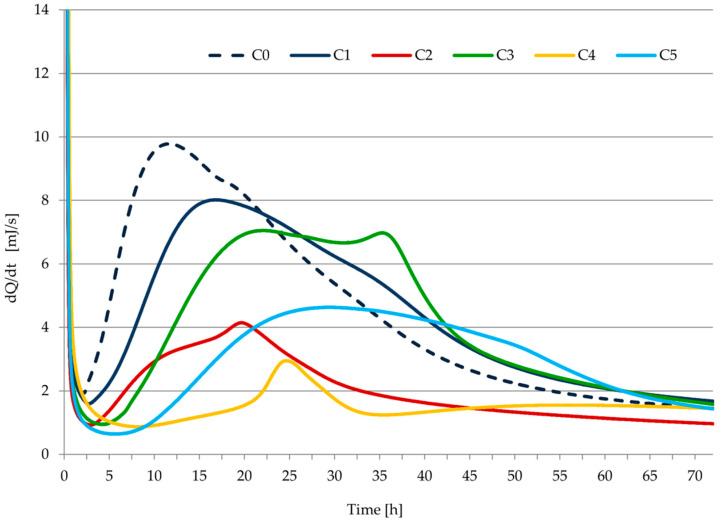
Rate of heat evolution as a function of time for all pastes used in the study.

**Figure 9:**
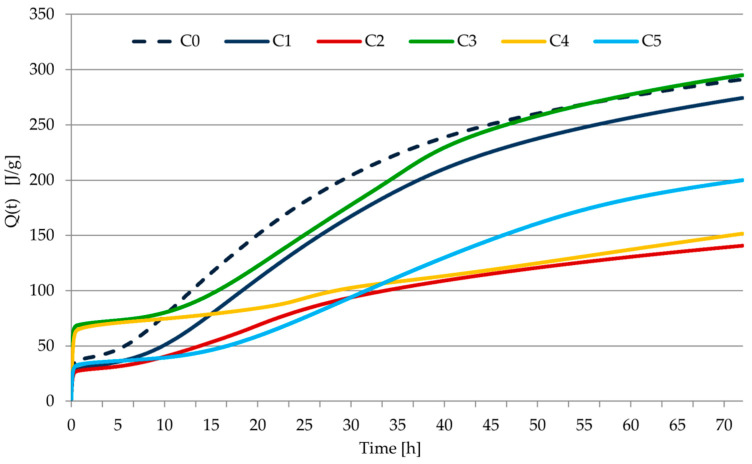
Total heat evolved as a function of time for all pastes used in the study.

The rate of heat evolution and the total heat released during the hydration of the tested pastes C1–C5 are shown in Figure 8 and Figure 9. Induction time and the total heat released by cement pastes after 12, 24, 36, 41, 48, 72 h of hydration are given in Table 10. The results of calorimetric measurements of cements modified with cellulose ether were supplemented with the results for the hydration heat of C0 paste (cement paste with CEM I without admixture). The microcalorimetric curves for cement paste containing CEM II/B-V 42.5 R show that both the total amount of evolved heat and the rate of heat evolution over time do not differ significantly, as compared to a base paste with cement CEM I 42.5 R. 

In the case of the C0 paste, the typical course of the heat release curve during the hydration of Portland cement is visible. During this process, the highest indications for the deepened heat effect related to the hydration of alite and tricalcium aluminate [13,43] were achieved, and at the same time the shortest induction period. Cellulose ether admixture caused an extension of the induction period and a delay and suppression of the main heat release peak. As a result, the amount of heat release during hydration was reduced. The use of mineral additions in the cement (fly ash and ground granulated blast-furnace slag) caused longer shifts in time and reduced the occurrence of the main thermal effect as well as extending the induction period. This is usually related to the reduction of the cumulative amount of heat released. The amount of exhausted heat exceeded that determined for the Portland cement samples (C0 and C1) only in the case of sample C3. It is related to the occurrence of an additional, clear effect with a maximum recorded after about 37 h of hydration. It can be explained by the formation of calcium silicates rich in silicon, resulting from the initiation of the pozzolanic reaction [12,13,38,43,44,45]. 

There is a clear division between samples made of 32.5 and 42.5 class cements (about 30% to 50% compared to the cumulative amount of heat released after a certain period of hydration time). In the case of sample C5, in which, as in sample C3, CEM II/B-V cement was used, but of a lower class, no separate thermal effect was observed, which can be identified with a pozzolan reaction. Instead, the main heat effect was significantly extended over time and had a less pronounced maximum value than can be observed in other tests. This can be explained by the overlapping of two thermal effects. In the case of the C4 sample, where apart from silica fly ash, it is present in the form of a filler, the limestone had the longest induction period with a short-lived heat effect and the lowest intensity. Such a course of the thermal curve can be explained by the cement dilution caused by a non-reactive material that did not emit heat during the test.

It should be noted that the biggest heat emission from the hydration process was obtained with the C3 paste, and in the case of the C3 mortar, the biggest strength parameters were achieved after 28 days, as well as the biggest bulk density. In contrast, the smallest heat emission from hydration was obtained with samples of C2 and C3 paste, while the smallest mechanical properties and bulk density were noted in the case of the C2 and C4 mortars. However, C2 mortar from CEM III cement was characterized by a significant (second after C3 mortar) increase in strength in the period between 7 and 28 days. This proves that the processes essential for the strength of the sample took place in a later period, not covered by calorimetric measurements. 

## 4. Conclusions

In the presented research, tests of consistency, bulk density, water retention value, mechanical properties of mortars and the heat of hydration pastes were performed using commercial cement CEM I, CEM II and CEM III. The possibility of using these binders as components of plaster mortars modified with a cellulose ether admixture was assessed.

Based on the experimental results presented in this paper, the following conclusions can be drawn:

- All mortars containing cellulose ether had a lower consistency (flow and cone penetration) than the cement mortar without admixture. The use of a polymer admixture was advisable due to the need to obtain a homogeneous, coherent material, with no visible signs of component segregation, characterized by high water retention. High water retention value is indicated for plastering mortars.
- The flow of plasters modified with cellulose ether admixture (C1, C2, C3, C5 samples) was in the range of 164 mm to 169 mm, and the cone penetration was in the range of 7.7 cm to 8.5 cm. The standard consistency of mortars modified with cellulose ether did not show significant differences with respect to mortars with CEM II and CEM III, except for mortar C4 with CEM II/B-M (V-LL) 32.5 R cement. Mortar with this binder showed the lowest flow (155 mm) and the lowest cone penetration (6.6 cm). In order to obtain the consistency as for the reference mortar, the water-cement ratio should be increased or use can be made of appropriate admixtures, the compatibility of which with cellulose ether should be tested early.
- No effect of the type of cement on the water retention value was noted. The WRV value for all plastering mortars modified with cellulose ether was about 99% (during the study period). All plasters were characterized by high water retention during the test period (the water retention values were greater than 94%).
- The smallest bulk density of mortars in a plastic state (1421 kg/m^3^, 1422 kg/m^3^) and, at the same time, the highest efficiency were achieved by plasters made with the use of cements CEM III/A 32.5 N-LH (mortar C2) and CEM II/B-M (V-LL) 32.5 R (mortar C4).
- The type of cement, in particular the amount of clinker and additives, and the class of cement, have a key influence on the mechanical properties of mortars. However, regardless of the type of binder used, all plasters met the standard requirements for compressive strength and can be classified in categories III and IV [42]. The compressive strength (after 28 days) for mortars was in the range from 6.03 MPa to 9.28 MPa. Plasters qualify for category III if their compressive strength after 28 days is in the range 3.5–7.5 MPa, and for category IV when their compressive strength is above 6.0 MPa.
- The increase in flexural and compressive strength of the tested mortars was different, depending on the type and amount of the additive/additives. Comparing the results of compressive strength (after 28 days) of the mortar C1 with CEM I cement with the results of tests of mortars with cement CEM II and CEM III, the compressive strengths of mortars C2-C5 were from 72% to 111% of the compressive strength of mortar C1 (8.38 MPa). The smallest result was obtained for mortar with CEM II/B-M (V-LL) 32.5 N (6.03 MPa), but the largest result was obtained for mortar with CEM II B/V 42.5 R (9.28 MPa). Similar conclusions can be drawn on the basis of calorimetric tests.
- All mortars modified with cellulose ether, regardless of the type of cement, according to the applicable standard requirements, can be classified as ordinary mortars of plastic consistency [34,36,40]. Their bulk density in plastic state is greater than 1300 kg/m^3^ and their flow is in the range 140–200 mm.
- These results indicate the possibility of using cement with mineral additives chosen for this research as a binder in plastering mortars. The selection of the appropriate cement gave similar and in some cases even better results than for plastering mortars in which Portland cement CEM I (without additives) was applied. The use of tested cements CEM II and CEM III did not lessen water retention, which is an important parameter in the case of plastering mortars.
- Considering the consistency determined in accordance with the standard [35] and the possible methods of application of the plasters, and taking into account the composition of mortars specified in the adopted composition, all materials were suitable for manual application, and additionally the mortar with CEM III/A 32.5 N-LH (besides cement CEM I 42.5 R) can be applied by a machine.

The next stage of this work will be the performance of other tests relating to the standards for plastering mortars, assessment of mechanical properties after more than 28 days and assessment of the microstructure of these plasters.

## Figures and Tables

**Table 1:** Chemical composition of cements.

Components	CEM I 42.5 R	CEM III/A 32.5 N-LH	CEM II/B-V 42.5 R	CEM II/B-M (V-LL) 32.5 R	CEM II/B-V 32.5 R
SiO_2_	20.14	23.96	27.3	20.0	28.75
Al_2_O_3_	4.88	6.74	10.9	6.9	11.32
Fe_2_O_3_	3.44	1.99	3.8	2.9	4.23
CaO	64.05	50.38	46.2	53.1	44.42
Na_2_O	0.16	0.33	0.4	0.3	0.48
MgO	1.61	4.28	1.7	1.5	2.14
SO_3_	2.71	1.48	2.6	2.8	2.38
Cl	0.025	0.037	0.1	0.1	0.065
K_2_O	0.61	0.54	1.4	1.0	1.59

**Table 2:** Physical and mechanical properties of cements.

Properties	CEM I 42.5 R	CEM III/A 32.5 N-LH	CEM II/B-V 42.5 R	CEM II/B-M (V-LL) 32.5 R	CEM II/B-V 32.5 R
Water requirement of normal consistency (%)	27.1	29.3	30.3	26.7	28.0
Initial setting time (min)	215	277	254	215	315
Specific surface area (cm^2^/g)	3942	4146	4433	4532	3424
28 days compressive strength (MPa)	58.8	51.3	55.2	41.6	40.8

**Table 3:** Mortar mix proportion of all samples.

Component (g)	C0	C1	C2	C3	C4	C5
	CEM I 42.5 R	CEM I 42.5 R	CEM III/A 32.5 N-LH	CEM II/B-V 42.5 R	CEM II/B-M (V-LL) 32.5 R	CEM II/B-V 32.5 R
Cement	450	450	450	450	450	450
Fine aggregate	1350	1350	1350	1350	1350	1350
Admixture	-	4	4	4	4	4
Water	315	315	315	315	315	315
w/c Ratio	0.7	0.7	0.7	0.7	0.7	0.7

**Table 4:** Consistency results for all mortars.

Symbol of Mortar	Flow ^1^ (mm)	Cone Penetration ^2^ (cm)
C0	205	12.9
C1	165	8.5
C2	169	8.0
C3	165	7.7
C4	155	6.6
C5	164	7.9

^1^ Consistency was determined in accordance with standard [34]. ^2^ Consistency was determined in accordance with standard [35].

**Table 5:** Water retention results.

Symbol of Mortar	WRV10 (%)	WRV30 (%)	WRV60 (%)
C0	86.5	79.7	73.5
C1	99.6	99.3	98.9
C2	99.6	99.3	99.0
C3	99.6	99.3	98.9
C4	99.5	99.3	98.9
C5	99.5	99.4	99.0

**Table 6:** Bulk density results.

Symbol of Mortar	Bulk Density in Plastic State (kg/m^3^)	Bulk Density in Hardened State (kg/m^3^)
C1	1434	1392
C2	1421	1375
C3	1513	1454
C4	1422	1377
C5	1455	1440

**Table 7:** Average strength of the mortars.

Sample Designation	Flexural Strength (MPa)			Compressive Strength (MPa)		
	2d	7d	28d	2d	7d	28d
C1	1.10	1.73	3.38	2.30	3.69	8.38
	(0.041) ^1^	(0.042)	(0.155)	(0.084) ^2^	(0.094)	(0.146)
C2	0.44	0.61	2.76	0.96	1.39	7.27
	(0.005)	(0.005)	(0.261)	(0.047)	(0.056)	(0.264)
C3	0.93	1.37	3.96	1.99	2.80	9.28
	(0.014)	(0.097)	(0.076)	(0.056)	(0.221)	(0.301)
C4	0.37	0.62	2.42	0.72	1.39	6.03
	(0.005)	(0.005)	(0.195)	(0.043)	(0.105)	(0.247)
C5	0.49	0.97	3.06	1.05	2.16	7.85
	(0.008)	(0.029)	(0.285)	(0.023)	(0.113)	(0.672)

^1^ Standard deviation of flexural strength measurements. ^2^ Standard deviation of compressive strength measurements.

**Table 8:** Additional information obtained on the basis of flexural strength test.

Sample Designation	Flexural Strength (%)			Strength Gain (MPa)	
	2d	7d	28d	2d to 7d ^1^	7d to 28d ^2^
C1	100	100	100	0.63	2.30
C2	40.00	35.26	81.66	0.17	2.32
C3	84.55	79.19	117.16	0.44	3.03
C4	33.64	35.84	71.60	0.25	2.05
C5	44.55	56.07	90.53	0.48	2.57

^1^ The difference in endurance between the 7th and the 2nd day of maturation. ^2^ The difference in endurance between the 28th and the 7th day of maturation.

**Table 9:** Additional information obtained on the basis of a compressive strength test.

Sample Designation	Compressive Strength (%)			Strength Gain (MPa)	
	2d	7d	28d	2d to 7d ^1^	7d to 28d ^2^
C1	100	100	100	1.39	6.08
C2	41.74	37.67	86.75	0.43	6.31
C3	86.52	75.88	110.74	0.81	7.29
C4	31.30	37.67	71.96	0.67	5.31
C5	45.65	58.54	93.68	1.11	6.80

^1^ The difference in endurance between the 7th and the 2nd day of maturation. ^2^ The difference in endurance between the 28th and the 7th day of maturation.

**Table 10:** Heat of hydration of pastes.

Symbol of Paste	Induction Period (h)	Heat after Hours of Hydration (J/g)					
		12	24	36	41	48	72
C0	2 h 21 min	93.18	174.97	226.97	241.32	256.61	291.22
C1	2 h 51 min	60.95	134.97	195.06	213.67	233.07	274.41
C2	3 h 5 min	45.26	80.65	103.57	110.33	118.60	140.67
C3	5 h 3 min	85.58	144.88	210.24	233.25	253.48	295.02
C4	8 h 10 min	76.14	90.81	109.19	114.37	122.42	151.60
C5	6 h 15 min	41.55	72.13	115.95	133.27	155.17	199.95

## Data Availability

No new data were created or analyzed in this study. Data sharing is not applicable to this article.
